# Pancreatic index: A prognostic factor of upfront surgery for body or tail pancreatic ductal adenocarcinoma with vascular involvement—A retrospective study

**DOI:** 10.1002/cam4.6687

**Published:** 2023-11-07

**Authors:** Lihan Qian, Jingfeng Li, Yanjun Sun, Weimin Chai, Xiaxing Deng, Weishen Wang, Baiyong Shen

**Affiliations:** ^1^ Department of General Surgery Pancreatic Disease Center, Ruijin Hospital Shanghai Jiaotong University School of Medicine Shanghai China; ^2^ Research Institute of Pancreatic Disease Shanghai Jiaotong University School of Medicine Shanghai China; ^3^ State Key Laboratory of Oncogenes and Related Genes Shanghai Jiaotong University Shanghai China; ^4^ Department of Cardiovascular Ruijin Hospital, Shanghai Jiao Tong University School of Medicine Shanghai China; ^5^ Department of Radiology Ruijin Hospital, Shanghai Jiao Tong University School of Medicine Shanghai China

**Keywords:** borderline resectable, locally advanced, pancreatic body/tail cancer, pancreatic ductal adenocarcinoma, pancreatic index

## Abstract

**Background:**

The pancreatic index (PI) is a useful preoperative imaging predictor for pancreatic ductal adenocarcinoma (PDAC). In this retrospective study, we determined the predictive effect of PI to distinguish patients of pancreatic body/tail cancer (PBTC) with vascular involvement who can benefit from upfront surgery.

**Method:**

All patients who received distal pancreatectomy for PDAC from 2016 to 2020 at the Pancreatic Disease Center, Ruijin Hospital, Shanghai Jiaotong University School of Medicine were considered for the study. A total of 429 patients with PBTC were assessed in relation to the value of PI. Fifty‐five patients were eventually included and divided into low PI group and 29 patients in the normal PI group.

**Results:**

The median overall survival (mOS) was significantly shorter in the low PI group (13.1 vs. 30.0 months, *p* = 0.002) in this study, and PI ≥ 0.78 (OR = 0.552, 95% CI: 0.301–0.904, *p* = 0.020) was an independent influencing factor confirmed by multivariate analysis. Subgroup analysis showed that PI was an independent prognostic factor for LA‐PBTC (OR = 0.272, 95% CI: 0.077–0.969, *p* = 0.045). As for BR PBTC, PI (OR = 0.519, 95% CI: 0.285–0.947, *p* = 0.033) combined with carbohydrate antigen 125 (CA125) (OR = 2.806, 95% CI: 1.206–6.526, *p* = 0.017) and chemotherapy (OR = 0.327, 95% CI: 0.140–0.763, *p* = 0.010) were independent factors.

**Conclusion:**

This study suggests that the PI can be used as a predictive factor to optimize the surgical indication for PBTC with vascular involvement. Preoperative patients with normal PI and CA125 can achieve a long‐term prognosis comparable to that of resectable PBTC patients.

## 1 INTRODUCTION

Pancreatic ductal adenocarcinoma (PDAC) is a lethal malignant tumor with an increasing worldwide incidence.^1^ Radical surgery remains the primary curative method for pancreatic cancer. Pancreatic body/tail cancer (PBTC) developing from the dorsal primordium was recently proven to be different from pancreatic head cancer (PHC) by different molecular profiles such as different lymphatic metastatic pathway.^2^, ^3^ PBTC usually involves more metastasis and less resectability than PHC at diagnosis.^4^, ^5^ Despite the adoption of neoadjuvant, the probability of resection is uncontrolled, and prognosis varies significantly for PBTC with vascular involvement.^6^ The effect of neoadjuvant therapy and the rate of receiving chemotherapy are lower in PBTC as well.^4^

The indications for PBTC have been optimized with surgical techniques.^7^, ^8^, ^9^ Vein reconstruction has been proven to be irrelevant to the long‐term prognosis of borderline resectable PBTC.^10^ Recently, distal pancreatectomy with en bloc celiac axis resection (DP‐CAR) has been performed by the worldwide high‐volume pancreatic surgery center for patients with locally advanced PBTC with tumors contacting the celiac axis.^10^, ^11^ However, early recurrence after the surgery was observed in that study, which was not predicted preoperatively by current clinically measured factors.^12^ In general, individual and specific surgical indications and strategies for PBTC are needed.

Pancreatic index (PI) is a useful preoperative imaging predictor for pathological changes reflecting the progression of local fibrosis in the pancreas, and it has been proved to be a strong independent predictor for PDAC basically resolved the influence of subcutaneous and visceral fat,^13^ and a decreased PI is relevant to precancerous lesions of PDAC.^14^

The aim of this study was to evaluate whether a decreased PI can be used to predict the prognosis of upfront surgery for PBTC with vascular involvement at a single institution.

## 2 MATERIALS AND METHODS

The study has been approved by the Ruijin Hospital Ethic Committee of Shanghai Jiaotong University of Medicine (RJ2020‐20), and the requirement for informed consent was waived due to the retrospective nature of the study. The study was in accordance with the Declaration of Helsinki. This retrospective cohort study is reported in line with the STROCSS 2021 guideline.^15^ The data collected were retrospectively controlled and classified.

### 2.1 Patients

All patients who received distal pancreatectomy (DP) for PDAC from 2016 to 2020 at the Pancreatic Disease Center, Ruijin Hospital, Shanghai Jiaotong University School of Medicine were considered for the study. A total of 429 patients were assessed according to the criteria listed below, and 84 patients were eventually included in the study.

Inclusion criteria: 1. Patients with PDAC located in the body/tail of the pancreas who underwent curative radical DP. 2. PDAC was diagnosed as borderline resectable (BR) according to the consensus proposed by the National Comprehensive Cancer Network without neoadjuvant chemotherapy, as follows: (a) superior mesenteric vein (SMV)/portal vein (PV)—tumor contact less than 180° or greater or bilateral narrowing/occlusion, not exceeding the inferior border of the duodenum; (b) celiac axis (CA)—tumor contact of less than 180° (abutment) without showing deformity/stenosis. 3. PDAC was diagnosed as locally advanced (LA) according to the following criteria: (a) CA—tumor contact greater than 180° (encasement) without contact with the proper hepatic artery (PHA), which makes distal pancreatectomy with en bloc celiac resection (DP‐CAR) possible; and (b) CHA—encasement without showing contact with the PHA or the presence of variant arterial anatomy, such as the right hepatic artery (RHA) or common hepatic artery (CHA), which makes DP‐CAR possible. 4 There was no evidence of distant metastasis according to preoperative images.

Exclusion criteria: 1. Distant metastases were confirmed intraoperatively. 2. Radical DP was not carried out, or total pancreatectomy was performed instead due to the unexpected progression of the tumor compared to preoperative image results. 3. Patients received neoadjuvant therapy. 4. Intraoperative freezing examination showed that the pancreatic transection margin was positive, and R0 resection was unrealized. Park W et al.^16^ proposed the recent definition of vessel abutment (<180°) or encasement (>180°). Finally, 84 patients were included and divided into the low PI group and normal PI group according to cutoff value (Figure S1). The details of vascular involvement were shown in Table S1.

### 2.2 Perioperative management and surgical procedure

Preoperative computed tomography (CT) was routinely performed for all patients. The diagnosis of the tumor, the identification of vascular involvement, and the surgical protocol were analyzed and determined by our multidisciplinary team, which consisted of the chief surgeon, first assistant, professor of anesthesiology, and at least two professors of radiology. The status of vascular involvement (encasement or abutment) was discussed, which was crucial for surgical indication. Vascular resection, reconstruction, and DP‐CAR were performed to ensure a curative operation through cooperation with the professor of cardiovascular surgery if necessary. The final surgical procedure was intraoperatively chosen by the chief surgeon who was well‐experienced (>100 cases) in undertaking a trial dissection. After discharge, all patients without contraindication were suggested to have a subsequent visit within 12 weeks after the operation, and their suitability for adjuvant chemotherapy was evaluated at the Department of Chemotherapy of our Pancreatic Disease Center. The primary chemotherapy regimens were categorized according to the Eastern Cooperative Oncology Group (ECOG) score as follows: ECOG 0–1 scores were treated with gemcitabine + capecitabine or FOLFIRINOX for really fit patients, while patients with ECOG 2 scores received gemcitabine mono.^17^, ^18^ Due to variations in postoperative recovery, individual reasons, economic factors, and regional differences, patients who eventually did not receive any chemotherapy were categorized as the “no adjuvant chemotherapy” group during follow‐up.

#### 2.2.1 Quantitative evaluation of the CT images

As previously shown, the PI was calculated by dividing the CT density of the pancreas by that of the spleen^13^ on plain CT. PI on arterial phase (PI‐a) was calculated by dividing the CT density of the pancreas by that of the spleen on contrast‐enhanced CT. Preoperative CT scans were regularly performed for each patient. Two individual radiologists who were blinded to the subjects' clinical information measured the mean pancreatic CT number for the largest possible spherical region of interest (ROI) of the pancreatic parenchyma at the resection line determined by the contrast of postoperative imaging with the first assistant if necessary. The mean splenic CT number was measured by tracing the ROI of the entire spleen at the level of the splenic hilum (Figure S2).

### 2.3 Follow‐up visit

After discharge, follow‐up was performed by telephone interviews every 2 months, recording the time and location of recurrence and patient survival. Disease‐free survival (DFS) and overall survival (OS) were calculated from the date of the operation to the date of tumor recurrence and death, respectively. Tumor recurrence and death were considered event data; no tumor recurrence or death was classified as censored data. Patients lost to follow‐up were classified based on the condition of the last follow‐up. Two patients who had postoperative 90‐day mortality were excluded. The follow‐up visits included 82 patients, excluding the two with 90‐day mortality. The rate of loss to follow‐up was 1.2%. Follow‐up visits ended in October 2022. The median follow‐up time was 16.7 months.

### 2.4 Statistical analysis

Statistical analyses were performed using SPSS statistical software (version 22). Continuous variables are expressed as means with standard deviation, as medians with range, or as rates (percentage). Continuous variables were compared using the Mann–Whitney *U* test. Categorical variables were compared using the chi‐square test and Fisher's exact test in case of small frequencies expected. The cutoff value of PI was determined by a time‐dependent receiver operating characteristic curve (ROC) using R language (version 4.2.1). For survival analysis, DFS and OS rates were analyzed by the Kaplan–Meier method and verified by the log‐rank test. For all tests, *p* values less than 0.05 were considered significant.

## 3 RESULTS

### 3.1 Patient characteristics and pathologic variables

There were 55 patients in the low PI group and 29 in the normal PI group included in this study. The ratio of BR (65.5% vs 89.7%) and LA (34.5% vs 10.3%) was significantly different between the low and normal PI groups (*p* = 0.016). The detailed classification of tumor involvement is shown in Table S1. The remaining demographic characteristics did not significantly differ between the two groups.

There were no significant differences in tumor diameter, surgical margin distance, number of positive lymph nodes, total retrieved lymph nodes, and proportion of chemotherapy between the two groups. The detailed data are shown in Table 1.

**TABLE 1:** Patient characteristics and pathologic variables between low and normal pancreatic index groups.

Characteristics	Low PI *n* = 55	Normal PI *n* = 29	*p* Value
Patient demographics			
Age, y	61 (44–89)	58 (28–73)	0.068
Sex, male	27 (49.1%)	20 (69.0%)	0.081
BMI, kg/m^2^	22.4 ± 2.8	22.1 ± 2.7	0.611
Smoking	11 (20.0%)	10 (34.5%)	0.145
Alcohol	44 (80.0%)	23 (79.3%)	0.940
Preoperative factors			
FBG	6.7 ± 2.5	6.7 ± 3.3	0.110
Hb, g/L	133 (101–166)	138 (104–168)	0.312
PLT, 10^9/L	182 (62–328)	160 (90–269)	0.085
ALB, g/L	41 (29–54)	44 (35–63)	0.078
CA199, U/mL	135.3 (0.8–9479.0)	254.6 (0.8–11,936)	0.286
CA125, U/mL	16.1 (6–195.2)	18.4 (6.3–111.5)	0.625
TB, μmol/L	18.2 ± 22.9	12.6 ± 3.4	0.093
BR/LA	36 (65.5%)/19 (34.5%)	26 (89.7%)/3 (10.3%)	0.016
Pathologic variables			
Tumor size, cm	3.82 ± 1.59	4.20 ± 1.61	0.351
Surgical margin distance, cm	1.12 ± 0.75	1.35 ± 0.84	0.427
N stage			
N0	19 (34.5%)	14 (48.3%)	0.221
N1	28 (50.9%)	11 (37.9%)	0.257
N2	8 (14.5%)	4 (13.8%)	1.000
Total retrieved LNs	10.0 ± 7.1	11.9 ± 8.1	0.298
No. positive LNs	1.6 ± 1.8	1.7 ± 2.3	0.811
Chemotherapy	35 (63.9%)	24 (82.8%)	0.068

Abbreviations: BR, borderline resectable; FBG, fasting blood glucose; LA, locally advanced; PI, pancreatic index.

### 3.2 Perioperative risk

Postoperative mortality, reoperation rate, complications, operation time, intraoperative bleeding, and intraoperative transfusion between the two groups were compared. In this study, two patients in the normal PI group experienced postoperative 90‐day mortality. One patient experienced acute cerebral infarction on the third day after the operation. He died on the 29th day after the surgery due to circulatory failure and central hyperpyrexia caused by massive cerebral infarction. The other patient had severe postoperative pancreatic exocrine insufficiency and died on postoperative Day 70 at home due to internal milieu disorder. Three patients in the normal PI group and one patient in the low PI group received unplanned reoperation. One patient in the normal PI group suffering grade C POPF received unplanned reoperation on postoperative Day 20 owing to postoperative hemorrhage, and the rest two patients in the normal PI group and one patient in low PI group without POPF underwent reoperation for postoperative hemorrhage caused by the stump of the splenic artery on postoperative Days 3, 4, and 8. Transcatheter endovascular embolization (TME) of the splenic arterial stump by DSA was successfully conducted on postoperative Day 17 in one patient in the low PI group suspected to have abdominal hemorrhage caused by grade C POPF. There were no statistically significant differences in the incidence of postoperative complications such as pancreatic fistula, intra‐abdominal abscess, intra‐abdominal bleeding, operative time, intraoperative bleeding, or transfusion. Details are shown in Table S2.

### 3.3 Tumor recurrence

The liver was the main site of tumor recurrence in both groups. The details of the other tumor recurrences are shown in Table S3, and there were no statistically significant differences.

### 3.4 Survival analysis

In total, 82 patients participated in follow‐up visits, excluding the two patients with 90‐day mortality. The cutoff value of PI was determined by a time‐dependent receiver operating characteristic (t‐ROC) curve, which showed the largest area under the curve (AUC) on postoperative Day 730 (Figure S3). ROC analysis was then carried out to find the cutoff at the greatest AUC of 2‐year postoperative survival (Figure S4). The cutoff value of PI‐a was calculated by the similar procedure; however, there was no statically diagnostic significance of PI‐a (*p* = 0.506). Univariate and multivariate analyses were performed to verify the statistical significance of the PI and PI‐a with well‐known clinical risk factors.

The median overall survival time (mOS) was significantly shorter in patients with PI < 0.78 (13.1 vs. 30.0 months, *p* = 0.002); mOS was significantly longer in patients with a normal range of CA125 (20.8 vs. 10.6 months, *p* = 0.000) and with chemotherapy (25.1 vs. 10.8 months, *p* = 0.000). Further multivariate analysis showed that PI ≥ 0.78 (odds ratio [OR] =0.552, 95% confidence interval (CI): 0.301–0.904, *p* = 0.020), CA125 (OR = 3.106, 95% CI: 1.701–5.673, *p* = 0.000), and chemotherapy (OR = 0.481, 95% CI: 0.288–0.802, *p* = 0.005) were independent factors of OS. The surgical approach of DP‐CAR is not an independent factor affecting the OS time (*p* = 0.109). We further compared patients with BR PBTC who underwent DP, and the result revealed that arterial abutment was not an independent factor affecting the OS time (*p* = 0.373) in this study. Details are shown in Table 2.

**TABLE 2:** Prognostic factors in univariate analysis (overall survival).

	Univariate analysis				Multivariate analysis		
	Patients (*n*)	mOS (month)	2‐YSR (%)	*p* Value	OR	95% CI	*p* Value
DP‐CAR, no/yes	60/22	21.1/12.9	45.0/18.2	0.109			
BR‐A, no/yes	24/36	23.8/19.0	47.2/37.5	0.373			
PI, <0.78/≥0.78/	55/27	13.1/30.0	25.5/63.0	0.002	0.552	0.301–0.904	0.020
PI‐a, <0.78/≥0.78/	23/59	14.1/18.4	39.1/35.6	0.884			
Age, <60/≥60 years	44/38	20.7/13.6	45.5/28.9	0.267			
ALB, <35/≥35 g/L	4/78	16.5/15.8	25.0/38.5	0.583			
TB, <24/≥24 μmol/L	76/6	16.5/12.5	36.8/50.0	0.424			
Preoperative CA19‐9, <37/≥37 (U/mL)	19/63	15.4/18.4	31.6/39.7	0.433			
Preoperative CA125, <35/≥35 (U/mL)	64/18	20.8/10.6	45.3/11.1	0.000	3.106	1.701–5.673	0.000
N stage, N0/N1&N2	32/50	15.8/16.5	40.6/36.0	0.611			
Intraoperative bleeding, <300//≥300 mL	50/32	12.7/14.1	17.5/19.0	0.285			
Intraoperative transfusion, no/yes	40/42	24/13.4	47.5/23.8	0.067	/	/	0.257
POPF, no/yes	55/27	16.5/16.9	34.5/37.0	0.900			
Adjuvant chemotherapy, no/yes	23/59	10.8/25.1	4.3/49.2	0.000	0.481	0.288–0.802	0.005

Abbreviations: 2‐YSR, 2‐year survival rate; BR‐A, borderline group with arterial abutment; DP‐CAR, distal pancreatectomy with en bloc celiac axis resection; mOS, median overall survival; PI, pancreatic index; PI‐a, pancreatic index on arterial phase; POPF, postoperative pancreatic fistula; TB, total bilirubin.

The median disease‐free time (mDFS) was significantly longer in patients with PI ≥ 0.78 (23.5 vs. 11.3 months, *p* = 0.015). Further multivariate analysis showed that PI ≥ 0.78 (OR = 0.462, 95% CI: 0.244–0.874, *p* = 0.018) was an independent factor of DFS. Similar to the univariate survival analysis for OS, we also compared the impact of DP‐CAR and arterial abutment on the DFS time. The results indicated that DP‐CAR (*p* = 0.206) and arterial abutment (0.164) were not independent factors affecting postoperative DFS time. Details are shown in Table 3.

**TABLE 3:** Prognostic factors in univariate and multivariate analyses (disease‐free survival).

	Univariate analysis			Multivariate analysis		
	Patients (*n*)	mDFS (m)	*p* Value	OR	95% CI	*p* Value
DP‐CAR, no/yes	60/22	12.7/23.5	0.206			
BR‐A, no/yes	24/36	14.1/7.6	0.164			
PI, <0.78/≥0.78	55/27	11.3/23.5	0.015	0.462	0.244–0.874	0.018
PI‐a, <0.78/≥0.78/	23/59	12.3/19.2	0.239			
Age, <60/≥60 years	44/38	12.3/17.2	0.430			
BMI, <22/≥22 kg/m^2^	23/59	12.7/14.1	0.760			
ALB, <35/≥35 g/L	4/78	6.4/13.4	0.260			
TB, <24/≥24 μmol/L	76/6	12.7/15.1	0.890			
Preoperative CA19‐9, <37/≥37 (U/mL)	19/63	17.5/12.7	0.950			
Preoperative CA125, <35/≥35 (U/mL)	64/18	14.1/11.9	0.090			
N stage, N0/N1&N2	32/50	13.4/12.7	0.925			
POPF, no/yes	55/27	13.4/14.1	0.740			
Intraoperative bleeding, <300//≥300 mL	50/32	15.1/11.9	0.317			
Intraoperative transfusion, no/yes	40/42	12.7/14.1	0.285			
Adjuvant chemotherapy, no/yes	23/59	14.3/12.7	0.725			

Abbreviations: BR‐A, borderline group with arterial abutment; CI, confidence interval; DP‐CAR, distal pancreatectomy with en bloc celiac axis resection; HR, hazard ratio; mDFS, median disease‐free survival time; PI, pancreatic index; PI‐a, pancreatic index on arterial phase; POPF, postoperative pancreatic fistula; TB, total bilirubin.

#### 3.4.1 Subgroup analysis

**FIGURE 1:**
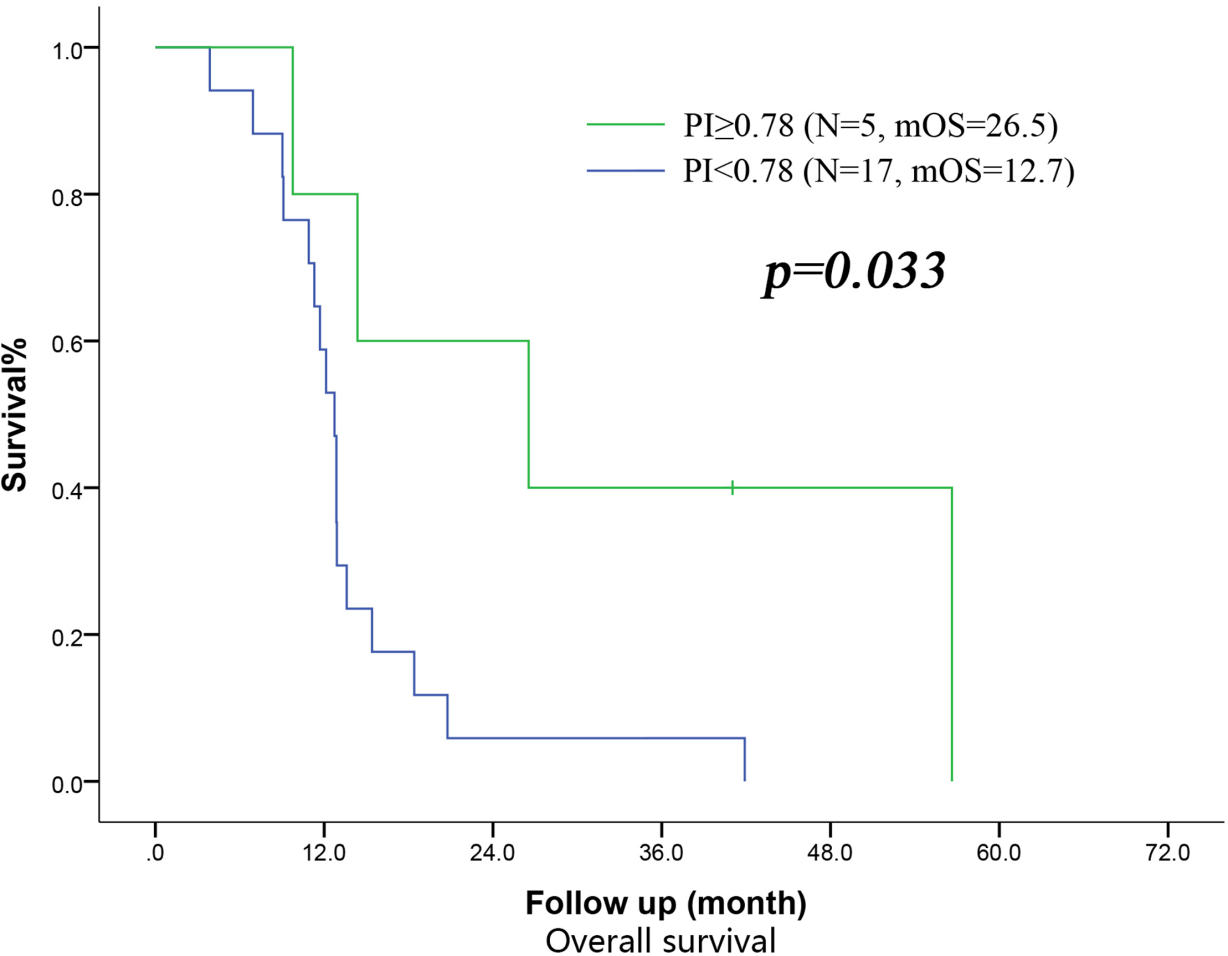
Survival curve of LA subgroup according to the PI (</≥0.78). mOS, median overall survival.

In the subgroup analysis of patients with LA‐PBTC, the OS time of patients in normal PI group was longer than that for those in low PI group (mOS: 26.5 months vs. 12.7 months, *p* = 0.033). Multivariate analysis confirmed that PI was an independent factor (OR = 0.272, 95% CI: 0.077–0.969, *p* = 0.045) (Figure 1). No other independent influencing factor of OS was found. There was no influencing factor proven independently relevant to the DFS time of LA‐PBTC.

**FIGURE 2:**
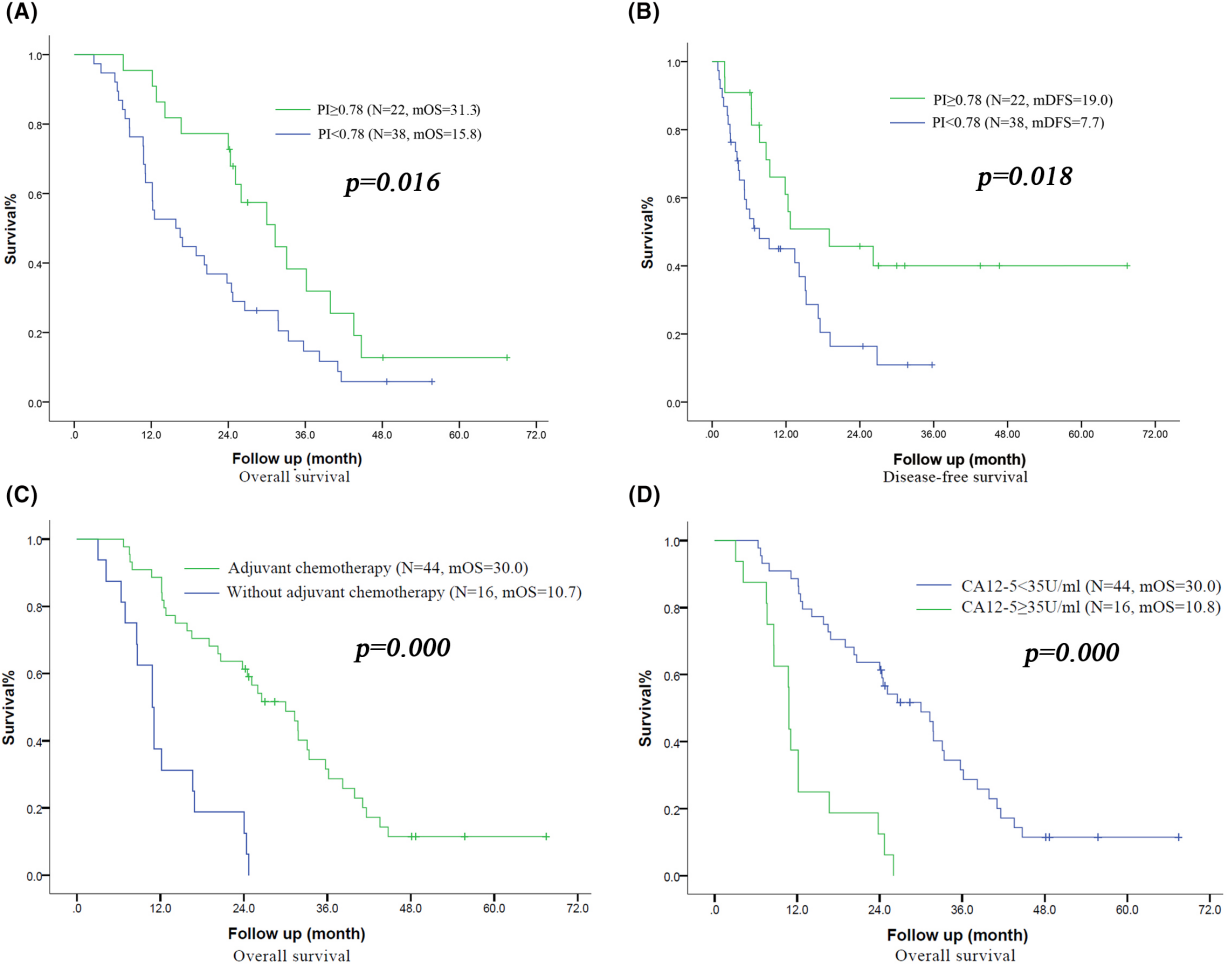
Survival curve of BR subgroup. A. Survival curve of OS according to the PI (</≥0.78), B. Survival curve of DFS according to the PI (</≥0.78), C. Survival curve of OS according to the adjuvant chemotherapy (±), D Survival curve of OS according to the CA125 (</≥35 U/mL). mDFS, median disease‐free survival; mOS, median overall survival.

In the subgroup of patients with BR PBTC, the OS time and DFS time of patients in the normal PI group were longer than those in the low PI group (mOS: 31.3 months vs. 15.8 months, *p* = 0.016, mDFS: 19.0 months vs. 7.7 months, *p* = 0.010). The mOS of patients who received chemotherapy (mOS: 30.0 months vs. 10.7 months, *p* = 0.000) and patients with CA125 < 35 U/mL (mOS: 30.0 months vs. 10.8 months, *p* = 0.000) were significantly longer. Further multivariate analysis showed that normal PI (OR = 0.519, 95% CI: 0.285–0.947, *p* = 0.033), CA125 ≥ 35 U/mL (OR = 2.806, 95% CI: 1.206–6.526, *p* = 0.017), and chemotherapy (OR = 0.327, 95% CI: 0.140–0.763, *p* = 0.010) were independent factors of OS (Figure 2).

## 4 DISCUSSION

The incidence of PDAC worldwide is projected to increase at an average annual growth of 1.1%.^19^ Cases of resectable PDAC remain low at 20% at diagnosis due to rapid tumor progression without obvious early clinical features.^20^ Unfortunately, a consideration proportion of patients suffer from short disease‐free and overall survival times due to lack of preoperative screening method. Approximately a quarter of patients with resectable tumors experience rapid relapse within 6 months after radical surgery.^21^ To our knowledge, there is currently no accessible preoperative evaluation method to screen patients with PBTC with early recurrence or poor prognosis, especially in cases with vascular involvement. It is thus necessary to evaluate the tumor condition and screen patients to optimize the treatment strategy before operating on DP or DP‐CAR for these targeted patients.

The diagnostic indicator, PI, which essentially eliminates the influence of individual differences in obesity was demonstrated to be a strong predictor for PDAC.^13^ The decrease in PI was closely related to local fatty degeneration and progression of fibrosis in the pancreas instead of visceral fat.^22^ Localized fat degeneration and intensified fibrosis are manifestations of local chronic inflammatory responses, accompanied by the infiltration of inflammatory cells which is crucial for the formation of an immunosuppressive tumor microenvironment.^23^ Pancreatic epithelial cells are prone to undergoing metaplasia and then dysplasia in a persistent inflammatory environment, eventually leading to the formation of in situ carcinoma. Moreover, tumor cells themselves stimulate pancreatic stellate cells, which further activate the degree of local fibrosis.^24^, ^25^ Especially for PBTC, tumor cells can spread through the main pancreatic duct to the rest of the pancreas. During this process, tumor cells might obstruct the pancreatic duct, leading to the activation of distant pancreatic stellate cells, thereby intensifying local inflammation and fibrosis. These mechanisms can all be reflected in the decrease of the PI value. Therefore, based on the results of this study and the aforementioned pathological foundation, to our acknowledge, this study is the first to propose preoperative PI should be utilized as a predictive factor to optimize the surgical indication for PBTC with vascular involvement who could benefit from upfront surgery.

Firstly, our results have unveiled that PBTCs with vascular involvement subjected to PI screening, irrespective of BR PBTC with arterial abutment, have attained a protracted survival akin to that observed in cases of resectable pancreatic cancer.^24^, ^25^ Recent studies prove that simple vessel abutment on preoperative imaging might not be a surgical contraindication or a sign of poor prognosis with recent surgical techniques^7^, ^26^, ^27^ which is consistent with our conclusion. These specific patients can benefit from upfront surgery, rather than risking tumor progression by receiving a uniform neoadjuvant treatment due to the lack of personalized regimens. An undeniable point to consider is that, imaging is currently the first‐line method for the indication of upfront surgery and neoadjuvant therapy.^9^ However, it remains challenging to evaluate treatment response and resectability and optimize individual indications for neoadjuvant therapy.^28^ The unexpected progression of local tumors and a lower resection rate after neoadjuvant therapy were inevitably observed in a previous study.^29^ In addition, evaluation of artery invasion by preoperative imaging is sometimes ambiguous due to the elasticity of the arterial wall, which may lead to overestimation of local invasion and delay the possibility of primary resection.^30^

Besides, PBTC differs from pancreatic head cancer in that, even when involving the CA, there is still an opportunity for DP‐CAR, whereas the latter is more prone to SMA invasion,^31^ making surgery unfeasible. In recent years, with the advancement of surgical techniques, an increasing number of centers have been performing DP‐CAR surgeries.^21^, ^22^, ^23^ Both previous literature^27^ and the results of this study have confirmed the safety of DP‐CAR. In this context, the findings of survival analysis of LA subgroup in this study further suggest that through PI screening, patients with high PI who undergo DP‐CAR treatment achieve long‐term survival similar to that of resectable pancreatic cancer. This emphasizes that even among PBTC cases involving CA, the tumor heterogeneity can lead to variations in the overall disease progression among patients.^32^ PI may serve as an effective indicator reflecting such differences.

In addition to the PI factor, in our subgroup analysis, we found that a high CA 125 level indicates a poorer prognosis for BR PBTC patients. CA 125 is an important marker that can help predict the outcomes for PDAC.^33^ These patients may not benefit from upfront surgery, even if it may involve only venous abutment. The biomarker CA 199 has been identified as adverse factor for pancreatic cancer prognosis^34^ and recent research further proposed that CA 19–9 levels can even predict lymph node metastasis, thereby predicting the effectiveness of upfront surgery for resectable pancreatic cancer.^35^ However, these two factors did not have a significant impact on patient prognosis in this study. We believe a possible explanation is that for BR PBTC patients, CA19‐9 levels overall are relatively close and may not be as sensitive as in early diagnosis of pancreatic cancer. CA125, on the other hand, exhibits higher sensitivity in predicting post‐surgery outcomes for this subgroup of patients. It is necessary to investigate and confirm the biomarkers, the status of lymph node metastasis, and other influencing factors, in conjunction with PI in future clinical trials to help predict the prognosis.

Alternatively, the predict value of PI‐a was unsatisfactory in our study. The intraindividual variability in pancreatic tissue which was caused by abdominal perfusion, cardiac status, and age on contrast‐enhanced CT could be an excuse as the previous study mentioned.^36^

However, this retrospective study also had some limitations. The sample size of the study needs to be expanded which led to selection bias. Second, our study is a single‐center study, and further multicenter clinical studies are needed to establish a more generalized baseline cutoff value. Besides, the predictive effect of PI for patients after neoadjuvant therapy should be verified by randomized controlled trials in future to optimize individual strategy based on the conclusion of this study. Our team aims to lay the theoretical foundation for future worldwide studies in various centers to investigate whether high PI patients benefit from neoadjuvant therapy by sharing the results of this study.

## 5 CONCLUSION

This retrospective study provides evidence that PI should be utilized as a predictive factor to determine the feasibility for upfront surgery for PBTC with vascular involvement. For patients with normal PI and CA125 levels, direct surgery followed by chemotherapy is a safe and personalized high‐quality treatment strategy.

## AUTHOR CONTRIBUTIONS

**Lihan Qian:** Conceptualization (lead); data curation (lead); formal analysis (equal); investigation (equal); methodology (equal); resources (lead); software (lead); validation (lead); writing – original draft (lead). **Jingfeng Li:** Data curation (equal); formal analysis (equal); resources (equal); validation (equal); writing – original draft (equal). **Yanjun Sun:** Data curation (equal); resources (equal); validation (equal); writing – original draft (equal). **Weimin Chai:** Data curation (equal); resources (equal); software (equal). **Xiaxing Deng:** Validation (equal); writing – review and editing (equal). **Weishen Wang:** Validation (equal); visualization (equal); writing – review and editing (equal). **Baiyong Shen:** Project administration (lead); writing – review and editing (lead).

## FUNDING INFORMATION

None.

## CONFLICT OF INTEREST STATEMENT

Lihan Qian, Jingfeng Li, Yanjun Sun, Weimin Chai, Xiaxing Deng, Weishen Wang, and Baiyong Shen have no conflicts of interest or financial ties to disclose.

## Supporting information


Figure S1.

Figure S2.

Figure S3.

Figure S4.

Tables S1–S3.

Data S1.

## Data Availability

I confirm that my article contains a Data Availability Statement even if no data is available (list of sample statements) unless my article type does not require one. I confirm that I have included a citation for available data in my references section, unless my article type is exempt.
