# Supplementary figures and images for: Pancreatic index: A prognostic factor of upfront surgery for body or tail pancreatic ductal adenocarcinoma with vascular involvement—A retrospective study
Source: Cancer Med. 2023 Nov 7;12(23):21199–208. doi: 10.1002/cam4.6687 (PMC10726763; doi:10.1002/cam4.6687)

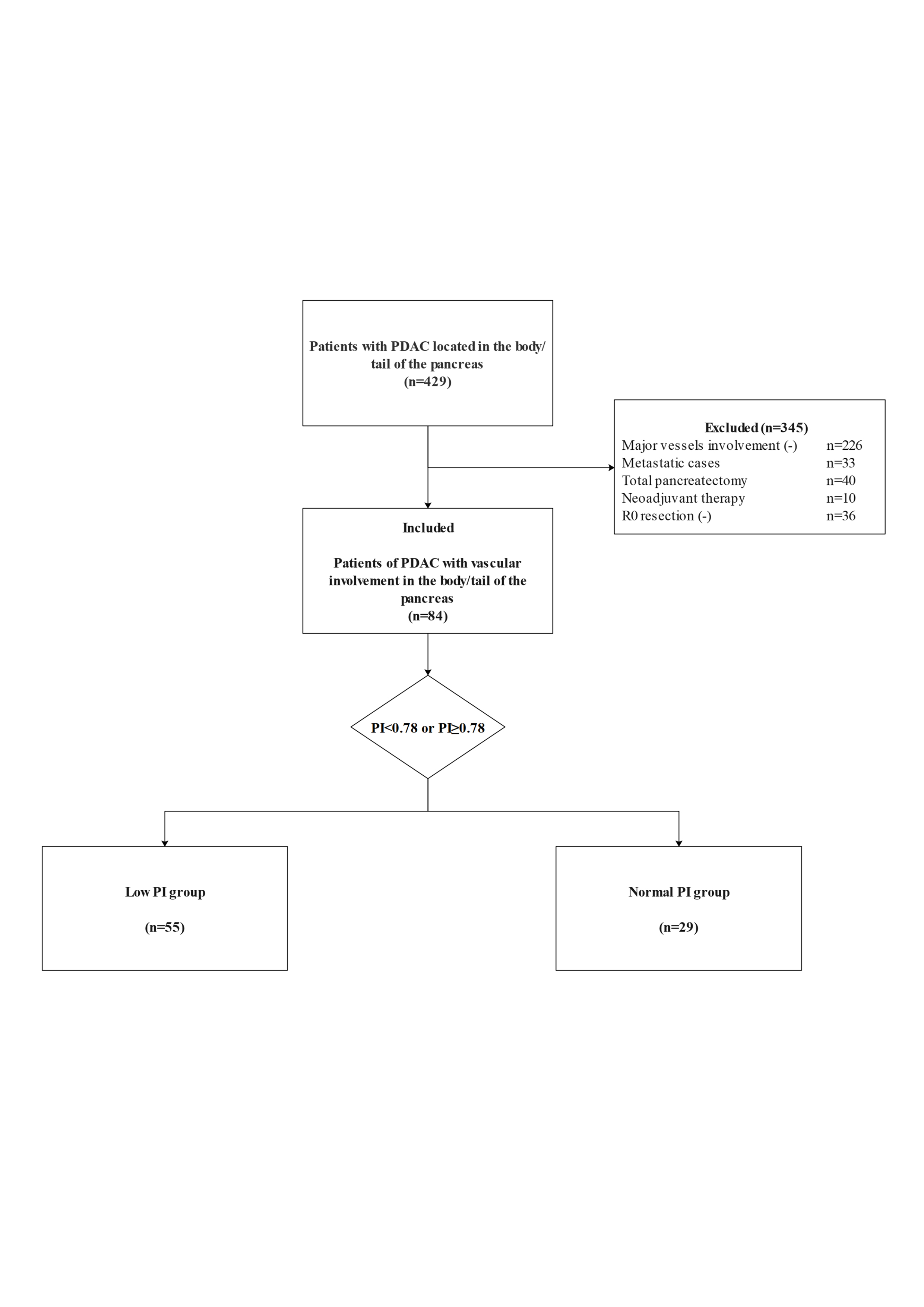

Supplement: Supplementary file 1 — Figure S1. [file CAM4-12-21199-s006.jpg]

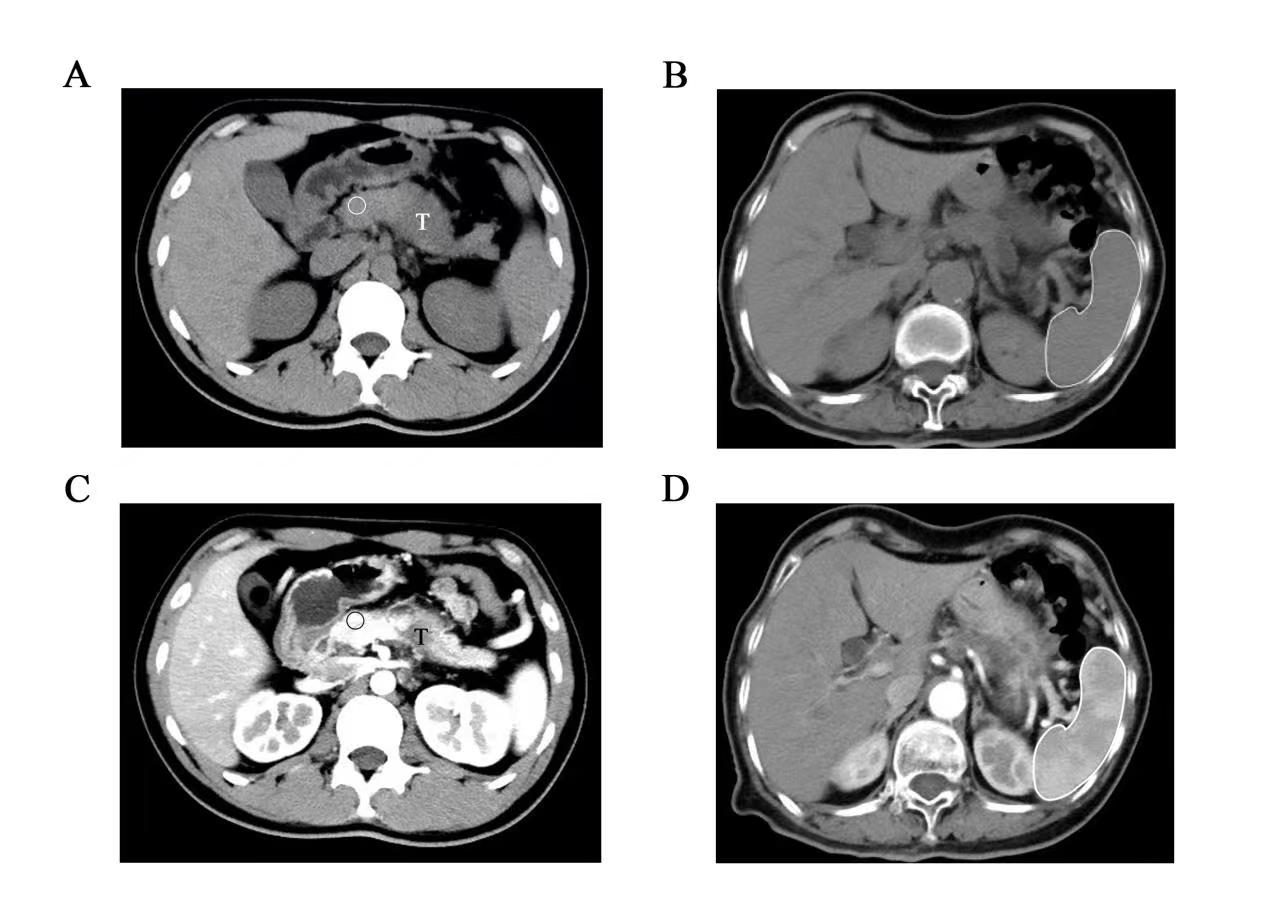

Supplement: Supplementary file 2 — Figure S2. [file CAM4-12-21199-s004.jpg]

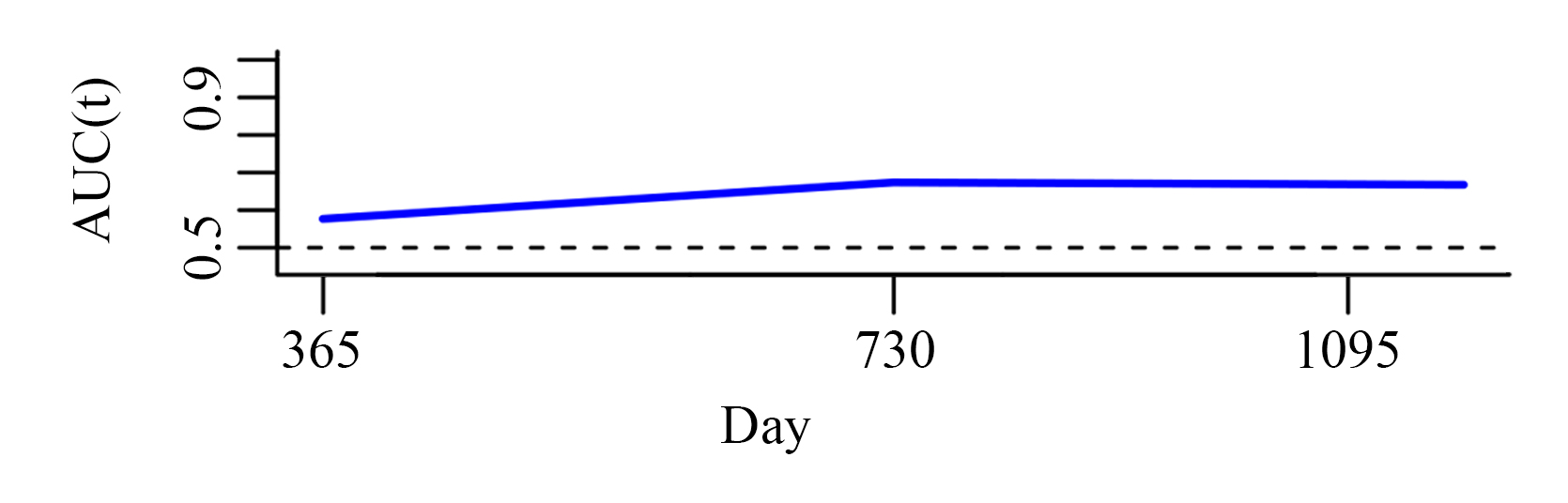

Supplement: Supplementary file 3 — Figure S3. [file CAM4-12-21199-s001.jpg]

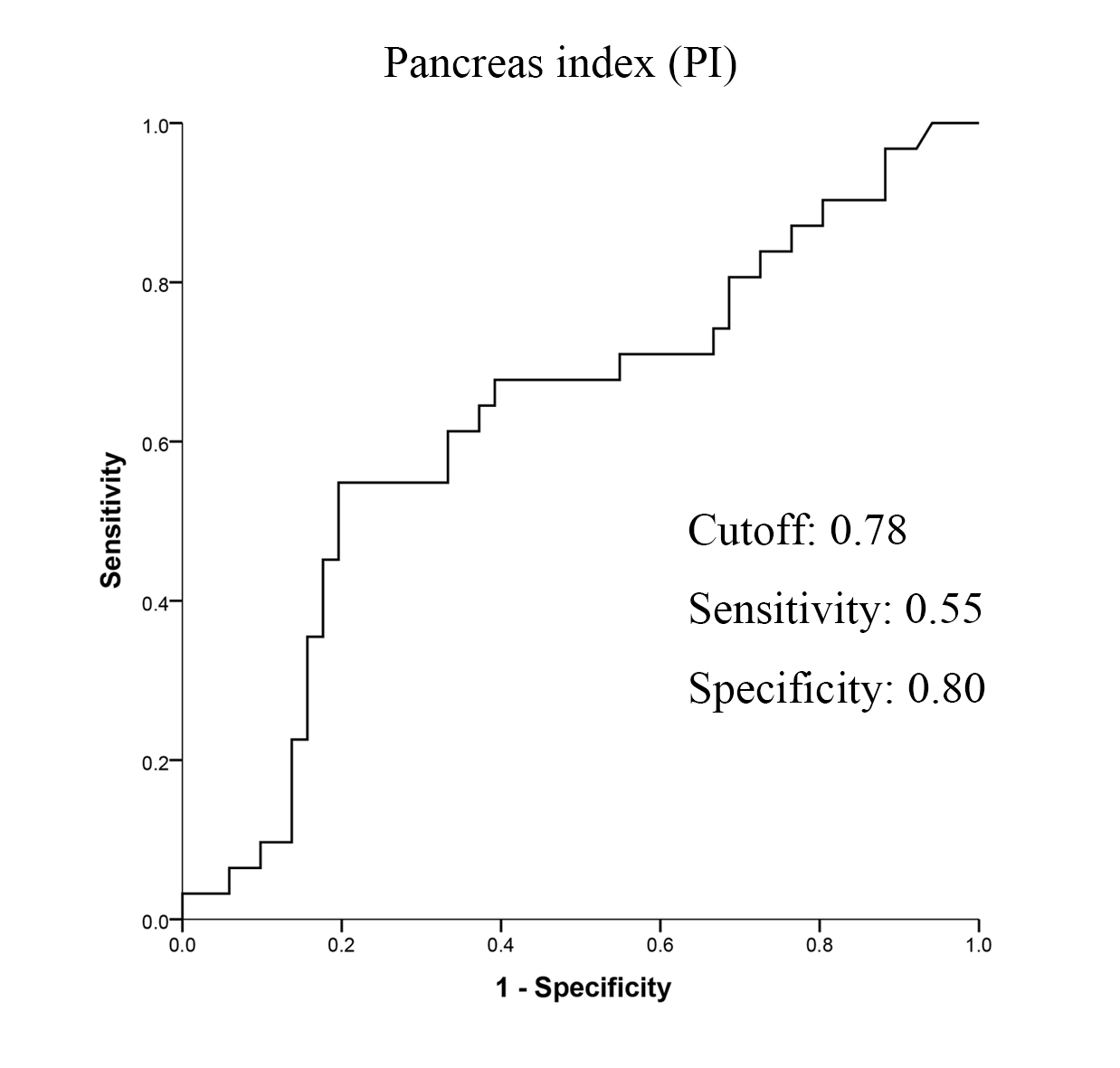

Supplement: Supplementary file 4 — Figure S4. [file CAM4-12-21199-s005.jpg]
