# Supplementary material for: Pancreatic index: A prognostic factor of upfront surgery for body or tail pancreatic ductal adenocarcinoma with vascular involvement—A retrospective study
Source: Cancer Med. 2023 Nov 7;12(23):21199–208. doi: 10.1002/cam4.6687 (PMC10726763; doi:10.1002/cam4.6687)
Supplement: Supplementary file 5 — Tables S1–S3. [file CAM4-12-21199-s003.docx]

**Supplemental table 1.** Classification of tumor involvement in details

| **Pathologic variables** | ***n*= 84** |
| --- | --- |
| Artery abutment |  |
| CHA^*^ | 12(14.3%) |
| CA | 36(42.9%) |
| Artery encasement |  |
| CHA^**^ | 9(10.7%) |
| CA | 22(26.2%) |
| Appleby^***^ | 22(26.2%) |
| Vein abutment | 20(23.8%) |
| Vein encasement | 17(20.2%) |
| Vein reconstruction | 17(20.2%) |

CHA common hepatic artery, CA celiac axis

^*^ 11 patients with artery abutment of both CHA and CA

^**^ 9 patients with artery encasement of both CHA and CA

^***^ Two patients who underwent Appleby surgery also received vein reconstruction intraoperatively.

**Supplemental table 2.** Perioperative risk and postoperative complications

|  | **Low PI**  ***n*=55** | **Normal PI**  ***n*=29** | ***P* value** |
| --- | --- | --- | --- |
| 90-day mortality | 0(0.0%) | 2(6.9%) | 0.223 |
| Reoperation | 1(1.8%) | 3(10.3%) | 0.228 |
| DSA | 1(1.8%) | 0(0.0%) | 1.000 |
| POPF |  |  |  |
| Grade B | 20(36.4%) | 6(20.7%) | 0.140 |
| Grade C | 1(1.8%) | 1(3.4%) | 1.000 |
| Intra-abdominal abscess | 14(25.5%) | 7(24.1%) | 0.895 |
| Intra-abdominal bleeding | 2(3.6%) | 3(10.3%) | 0.453 |
| Postoperative chyle leakage | 0(0.0%) | 1(3.4%) | 1.000 |
| Operation time, min | 200(120-480) | 240(120-370) | 0.428 |
| Intraoperative bleeding, ml | 300(50-3000) | 300(100-1500) | 0.215 |
| Intraoperative transfusion | 28(50.9%) | 16(55.2%) | 0.710 |

**Supplemental table 3.** Recurrence pattern

|  | **Low PI**  ***n*=55** | **Normal PI**  ***n*=27** | ***P* value** |
| --- | --- | --- | --- |
| Recurrence |  |  |  |
| Residual pancreas | 1(1.8%) | 1(3.7%) | 1.000 |
| Stomach | 2(3.6%) | 1(3.7%) | 1.000 |
| Liver | 15(27.3%) | 12(44.4%) | 0.120 |
| Lung | 3(5.5%) | 1(3.7%) | 1.000 |
| Bone | 1(1.8%) | 0(0.0%) | 1.000 |
| Peritoneal seeding | 7(12.7%) | 4(14.8%) | 1.000 |
| Retroperitoneal lymph nodes | 2(3.6%) | 1(3.7%) | 1.000 |
