# Supplementary material for: Pancreatic index: A prognostic factor of upfront surgery for body or tail pancreatic ductal adenocarcinoma with vascular involvement—A retrospective study
Source: Cancer Med. 2023 Nov 7;12(23):21199–208. doi: 10.1002/cam4.6687 (PMC10726763; doi:10.1002/cam4.6687)
Supplement: Supplementary file 6 — Data S1. [file CAM4-12-21199-s002.docx]

**Supplemental figure 1** Flow chart of inclusion. Patients divided in the low PI and normal PI group accordingto the PI.

**Supplemental figure 2** Plain and contrast-enhanced CT analysis of PI. A. Preoperative plain CT images of thepatient with PBTC. The mean pancreatic CT number was measured for the spherical range of interest (ROI) of the pancreatic parenchyma at the estimated resection plan. B. The mean splenic CT number was measured by tracing the ROI of the entire spleen at the level of the splenic hilum. C.D. Preoperative contrast-enhanced CT images of the patient with PBTC in arterial phase. The mean pancreatic and splenic CT number and were measured by the same method as mentioned before. The PI was calculated as the ratio of the CT number of the pancreas to the CT number of the spleen on plain and contrast-enhanced CT.

**Supplemental figure 3** Time-dependent ROC curve. The greatest AUC was 0.642 on postoperative Day 730, and AUCs on postoperative Days 365 and 1095 were 0.594 and 0.554, respectively.

**Supplemental figure 4** ROC curve of the pancreatic index for 2-year postoperative survival, with AUC=0.642, p=0.049
